# Trauma, post-traumatic stress disorder and violence in the prison population: prospective cohort study of sentenced male prisoners in the UK

**DOI:** 10.1192/bjo.2022.639

**Published:** 2023-03-03

**Authors:** Emma Facer-Irwin, Nigel Blackwood, Annie Bird, Deirdre MacManus

**Affiliations:** Department of Forensic and Neurodevelopmental Sciences, Institute of Psychiatry, Psychology and Neuroscience, King's College London, UK; HMP Wandsworth, South London & Maudsley NHS Foundation Trust, UK; and Department of Forensic and Neurodevelopmental Sciences, Institute of Psychiatry, Psychology and Neuroscience, King's College London, UK; HMP Wandsworth, South London and Maudsley NHS Trust, UK; Department of Forensic and Neurodevelopmental Sciences, Institute of Psychiatry, Psychology and Neuroscience, King's College London, UK; and London and South East NHS Veterans’ Mental Health Service, Camden and Islington NHS Trust, UK

**Keywords:** Forensic mental health services, post-traumatic stress disorder, trauma, prison, violence

## Abstract

**Background:**

Violence is a common problem in prisons. Post-traumatic stress disorder (PTSD), a prevalent disorder in prison populations, has been identified as a risk factor for violent behaviour in community and military populations. Although cross-sectional associations between PTSD and prison violence have been documented, prospective cohort studies are required.

**Aims:**

To investigate whether PTSD is an independent risk factor for prison violence, and examine the potential role of PTSD symptoms and other trauma sequelae on the pathway from trauma exposure to violent behaviour in prison.

**Method:**

A prospective cohort study was conducted in a large, medium security prison in London, UK. A random sample of sentenced prisoners arriving into custody (*N* = 223) took part in a clinical research interview, which assessed trauma histories, mental disorders including PTSD, and other potential sequelae of trauma (anger, emotion dysregulation). Incidents of violent behaviour were measured with prison records covering the 3 months after reception into custody. Stepped binary logistic regression and a series of binary mediation models were performed.

**Results:**

Prisoners who met current (past month) criteria for PTSD were more likely to engage in violent behaviour during the first 3 months of imprisonment, after adjusting for other independent risk factors. The relationship between lifetime exposure to interpersonal trauma and violent behaviour in custody was mediated by total PTSD symptom severity. Hyperarousal and negatively valenced cognitive and emotional appraisal symptoms were particularly implicated in this pathway.

**Conclusions:**

The identification and treatment of PTSD has the potential to reduce violence in prison populations.

Violence in prison is a common problem,^1^ with a significant deleterious impact on prisoner and staff group physical and mental health.^2,3^ Imprisoned populations report high rates of exposure to traumatic experiences such as child abuse and interpersonal violence,^4^ and have higher rates of post-traumatic stress disorder (PTSD)^5^ compared with community samples. Across the empirical literature, exposure to interpersonal violence has been identified as one of the strongest predictors for future violent behaviour.^6^ An increased risk of violent behaviour among those with PTSD has also been demonstrated in community and military populations.^7–10^ A recent systematic review^11^ identified cross-sectional associations between PTSD and violent behaviour in prison populations. However, heterogeneity of the included studies precluded the use of meta-analytic techniques to provide summary estimates of the strength of the observed association, and no study examined associations between PTSD and violence with a prospective cohort design to attempt to establish causality. Anger and emotion dysregulation have also been implicated in pathways to prison violence.^12,13^ Understanding the role of past traumatic experiences and current PTSD in the prediction of prison violence is central to the development of trauma-informed care^14,15^ and the prevention of future harm. The present study thus had two aims: to investigate whether PTSD is an independent risk factor for prison violence, and to examine the potential role of PTSD symptoms and other trauma sequelae on the pathway from trauma exposure to violent behaviour in custody.

## Method

### Study design

The data were collected as part of a wider prospective cohort study examining the impact of PTSD on behavioural outcomes among a male sentenced prisoner population in London, UK. This paper reports on data collected at two time points: at time point 1, data was collected by clinical interviews within 2 weeks of arrival into prison custody after sentencing; at time point 2, data was collected by inspecting prison records for the 3-month period following reception.

### Sample

The study sample consisted of sentenced male prisoners aged 18–55 years who arrived into custody in the week before sampling at a large (prisoner capacity of 1650), category B (medium security) prison in London, UK. For a detailed description of recruitment processes and exclusion criteria, please see the recruitment flowchart in the Supplementary Material available at https://doi.org/10.1192/bjo.2022.639. Sampling occurred weekly between 1 July 2017 and 1 March 2019. Potentially eligible participants were identified from prison reception lists: of the 9075 prisoners received into custody over the data collection period, 38% (*n* = 3477) met initial eligibility criteria. A subgroup was randomly selected by a random number generating process, and approached by a researcher on the prison wings to obtain informed consent (*n* = 432). Of those approached, 20% declined to participate and a further 18% were excluded following approach (for example, because they were unable to communicate in English, give informed consent or considered too unsafe to be seen alone by a researcher because of aggression). The final sample size was *N* = 223.

Demographic data (ethnicity, age, main offence) was gathered from prison reception lists to identify differences between individuals who participated and those who declined to participate in the study, and to weight analyses for any potential non-response biases. Preliminary analysis indicated that participants did not differ from non-participants in terms of ethnicity (*χ*^2^ = 0.03, *P* = 0.864), age (*t* = −0.083, *P* = 0.41) or commission of a violent index offence (*χ*^2^ = 0.05, *P* = 0.82).

### Procedure

Details of recruitment and data collection procedures have been previously reported.^16^ The authors assert that all procedures contributing to this work comply with the ethical standards of the relevant national and institutional committees on human experimentation and with the Helsinki Declaration of 1975, as revised in 2008. All procedures involving human patients were approved by NHS England (approval number 16/SS/0179) and the National Offender Management Service (approval number 2016–321).

Written informed consent was obtained from all participants. Consenting participants then took part in a clinical interview with a researcher within 2 weeks of arrival into prison custody. All interviewers were postgraduate-level researchers in psychology (Masters level or above), with several years of experience working in forensic settings. As literacy and educational attainment levels in prison are often low, all self-report questionnaires included in this assessment were administered in an interview format. All interviewers received specific training in the administration of each of the tools, involving several weeks of observation, followed by practice and *in vivo* training sessions led by a senior consultant psychiatrist (D.M.) and/or the lead researcher (E.F.-I.) before data collection. Initial assessments were observed by the lead researcher and further spot checks conducted throughout the project for quality assurance and standardisation purposes. All measures were administered once only. Follow-up data on violent behaviour was then collected by researchers at 3 months post-reception into custody, using recorded incidents.

### Measures

#### PTSD

A diagnosis of PTSD (past month and lifetime) was established with the Structured Clinical Interview for DSM-5 (SCID-5),^17^ which has been frequently used in prison research. In the present study, the SCID-5 was found to have good interrater reliability (*κ* = 0.80). A continuous measure of PTSD symptoms, the PTSD Checklist for DSM-5 (PCL-5),^18^ was also used to derive symptom clusters and facilitate mediation analyses. According to this instrument, PTSD symptoms are clustered into four main groups: re-experiencing (cluster B), avoidance (cluster C), negative alterations in cognitions or mood (cluster D) and hyperarousal (cluster E). The Cronbach's alpha score for the PCL-5 in the current study was 0.95, indicating good scale reliability and internal consistency.

#### Violent behaviour

The primary outcome of interest was violent behaviour, measured dichotomously at 3 months following reception, using recorded incidents documented on both the Computer-National Offender Management Information System (C-NOMIS) and a locally developed prison violence reduction database. Consistent with previous research,^7,19^ our definition of violence included incidents of physical violence (threatened or actual), fashioning or possession of weapons, verbal aggression and abusive behaviour, arson or other violent behaviour (e.g. hostage taking, rioting, barricading, violent damage to cells or property).

#### Interpersonal trauma exposure

Considering that exposure to previous violence is a strong predictor of both PTSD and future violence and aggression, interpersonal trauma exposure was selected as our proposed independent variable in mediation analysis. This variable was defined as cumulative lifetime exposure to interpersonal violence, which was measured at interview with the Life Events Checklist.^20^ Although much previous research has focused primarily on the role of childhood maltreatment in predicting later violent or criminal behaviour,^6^ the inclusion of interpersonal violence exposure extending into adulthood is also supported by research,^21,22^ and allowed for other forms of violence exposure – particularly those relating to community and gang-related violence – to be captured in our analysis. Events assessed and coded as involving interpersonal violence included physical assaults, assaults with weapons, sexual assaults, any other uncomfortable sexual experience, unlawful captivity and combat or exposure to a war zone. In this examination and in line with past prison research,^12^ exposure to interpersonal violence included both events directly experienced and witnessed happening to someone else, resulting in a total possible range of 0 to 12 events endorsed. Direct experience of traumatic incidents and the witnessing of the same were weighted equally. The total number of traumatic experiences endorsed by the individual was then used as a continuous measure of the experience of interpersonal trauma.

#### Clinical covariates

Other psychiatric disorders identified from literature reviews as risk factors for violence, and included and adjusted for in regression analyses, were substance misuse, alcohol misuse and dependence, psychosis, depression, mania, attention-deficit hyperactivity disorder (ADHD), antisocial personality disorder (ASPD) and borderline personality disorder. Probable substance misuse was measured with the Drug Abuse Screening Test, using a recommended cut-off score of ≥6.^23^ Harmful alcohol use and alcohol dependence were measured with the Alcohol Use Disorders Identification Test, using cut-off scores of 16–19 and ≥20, respectively.^24^ Depression was assessed with the Patient Health Questionnaire-9,^25^ with a severe symptom cut-off (≥15) used to indicate ‘probable’ diagnosis. ADHD was established by the Adult ADHD Self-Report Scale, a six-item self-report scale developed by the World Health Organization, with probable ADHD estimated using threshold cut-off scores for each symptom question.^26^ Mania, psychosis and ASPD were all assessed by the Mini International Neuropsychiatric Interview (MINI),^27^ a structured diagnostic interview used frequently in previous prison studies.^19^ As the MINI does not include a measure of borderline personality disorder, this was assessed with the SCID-5^17^ BPD module, introduced mid-way through data collection and measured on a subsample of the total population (*n* = 101). ASPD and BPD were found to be highly comorbid within our sample: all except for three individuals with BPD also had comorbid ASPD. As assessing BPD individually in regression models would have restricted analysis in that group, and collinearity prevented these diagnoses from being assessed in the same model, a cluster B personality disorder category was created, whereby 1 indicated ASPD and/or BPD and 0 indicated neither diagnosis. No participants in the sample met current criteria for mania, and so this psychiatric comorbidity was dropped from subsequent analysis. Missing data was present for some variables examined in this analysis: ADHD (*n* = 3), cluster B personality disorder (*n* = 5), psychosis (*n* = 5).

#### Sociodemographic and forensic covariates

Sociodemographic data (age, ethnicity, highest qualification) was collected with a brief questionnaire developed for the purposes of the study. We had no access to historical conviction data from police databases (Police National Computer database). Thus, forensic information gathered as part of this questionnaire included self-reported offence history and prior history of imprisonment. A prisoner's current conviction, prisoner status (i.e. newly sentenced, transferred or recalled), sentence length and any gang affiliation was collected from prison records. Gang affiliation (1 indicates present, 0 indicates none recorded) was recorded on C-NOMIS as part of security assessments done by prison staff at initial reception. Violent offences were defined as: murder, attempted murder, assault, robbery, arson, any sexual offence (rape, sexual coercion, sexual harassment, child molestation), intimidation and making illegal threats.^28^ All other offences were categorised as ‘non-violent’ offences.

#### Proposed mediating variables

Potential mediators of the association between interpersonal violence exposure and violence in custody included PTSD symptoms; anger, assessed with the Dimensions of Anger Scale (DAR);^29^ and emotion dysregulation, measured with a brief version of the Difficulties in Emotion Regulation Scale (DERS-SF).^30^ Cronbach's alpha for both latter questionnaires was 0.88. The anger (*n* = 6) and emotion dysregulation (*n* = 5) variables contained missing data because of incomplete interviews (e.g. prisoner being moved or transferred before interview completion).

### Statistical analysis

Potential risk factors for prison violence were first examined with univariate analyses. Binary logistic regression was performed to examine whether PTSD at baseline was a risk factor for subsequent violence in custody. Other risk factors for violence that were also examined included sociodemographic (age, ethnicity, educational qualification, time at risk), forensic (sentence length, previous time in custody, gang affiliation, previous violent conviction) and clinical (substance misuse, harmful alcohol use/dependence, depression, psychosis, cluster B personality disorder, ADHD) covariates. A multivariate model that adjusted for all significant covariates was then performed.

To investigate our second research question regarding the role of post-traumatic stress symptoms or other trauma-related difficulties (anger, emotion dysregulation) on the pathway between interpersonal trauma exposure and violence in custody, a series of mediation models were then conducted. Bivariate (Pearson's *r*) correlations were first performed to investigate relationships between predictor (interpersonal trauma exposure) and proposed mediator variables (PTSD, anger, emotion dysregulation). Interaction effects between our proposed exposure and other included covariates were then assessed with the likelihood ratio test. Finally, mediation was performed with a binary mediation programme available through Stata for Windows (version 15.1). Examination of the coefficients and bootstrapped (1000 iterations) confidence intervals allowed the determination of the presence of any indirect effects.

## Results

### Sample characteristics

Participant characteristics (*n* = 223) are summarised in Table 1. The sample was ethnically diverse, and with no significant differences in age (*χ*^2^ = 1.6, *P* = 0.452) or ethnicity (*χ*^2^ = 5.3, *P* = 0.70) between our sample and the wider prison population, as reported in 2018.^31^ Our sample did differ from the wider prison population in that it included a smaller proportion of foreign nationals (14.5 *v*. 37.7%, *P* < 0.0001).^31^ The index offence(s) of our sample could not be compared because of a lack of reported data from the wider prison estate.

**Table 1:** Sample demographics

Characteristic total (*N* = 223)	Mean (s.d.) or *n* (%)
Age, years	31.3 (9.0)
Ethnicity	
White British	87 (39.4)
White other	35 (15.7)
Black African/Black Caribbean	57 (25.6)
South Asian	16 (7.2)
Mixed/other	28 (12.5)
Nationality	
UK	189 (85.1)
Foreign national (including EU)	33 (14.9)
Employment status at point of entry to prison	
Employed	106 (47.5)
Unemployed	117 (52.5)
Highest qualification^a^	
None	71 (32.4)
Any (GSCE/A level, degree, certificate)	149 (66.8)
Age upon leaving school, years	15.4 (2.5)
Relationship status	
Single	186 (83.4)
Married/cohabiting	37 (16.6)
Living situation (before prison)	
Fixed accommodation (including family)	177 (79.4)
No accommodation	46 (20.6)
Previous diagnosis of mental disorder	136 (61)
Index offence	
Violence against the person (e.g. actual or grievous bodily harm, murder)	55 (24.8)
Weapons	16 (7.2)
Theft/handling	40 (18)
Burglary	27 (12.3)
Robbery	10 (4.5)
Drugs	21 (9.5)
Fraud/forgery	15 (6.8)
Sexual offences	10 (4.5)
Other (e.g. motoring, breach of court order)	21 (9.5)
Previous history of violent offending	145 (65)
Sentence length >18 months^a^	70 (33.6)
Prisoner status^a^	
Newly sentenced	121 (55.3)
Transferred from other prison	57 (25.8)
Recalled to prison (license breach)	43 (19.5)

Demographic information for a sample of male sentenced prisoners (*N* = 223) residing in a large category B prison in south London. Demographic information assessed by a self-report questionnaire. Forensic information (i.e. index offence, sentence length, prisoner status) gathered from prison records. History of violent offending measured with a self-report questionnaire.

a. Sample size <221 because of missing data.

### PTSD as a risk factor for violence in custody

By the 3-month follow-up, 23% of the sample had engaged in at least one incident of violent behaviour in prison. Within this violent group (*n* = 52), 79% had just one recorded incident of violence within the follow-up period, with the frequency of violent incidents perpetrated by individuals ranging from 1 to 29.

PTSD and a range of other covariates were found to be predictive of violence in univariate analyses (see Table 2). In the multivariate model, the only factors that remained independently significantly associated with violence in custody were age, time at risk (i.e. number of days in custody), having previously been imprisoned, having gang affiliations and a current PTSD diagnosis. No other clinical covariates were identified as predictors of prison violence in univariate and multivariate analyses.

**Table 2:** Post-traumatic stress disorder as a risk factor for prison violence after adjustment for relevant covariates

Variable	Violent (*n* = 52)	Non-violent (*n* = 171)	Odds ratio	95% CI	Adjusted odds ratio^a^	95% CI
	Mean (s.d.) or *n* (%)	Mean (s.d.) or *n* (%)				
Demographic						
Age	28 (8.6)	33 (8.9)	0.9	0.9–0.98	0.9	0.9–0.98
BME (*n* = 101)	29 (28.7)	72 (71.3)	1.7	0.9-3.2		
White (*n* = 122)	23 (18.8)	99 (81.2)				
No qualifications (*n* = 73)	23 (31.1)	51 (68.9)	1.9	0.99–3.6		
Any qualifications (*n* = 148)	29 (19.5)	120 (80.5)				
Time at risk (days in custody)			1.04	1.02–1.06	1.04	1.02–1.1
Forensic						
Previous imprisonment (*n* = 170)	46 (27.1)	124 (72.9)	2.9	1.2–7.3	3.7	1.3–10.4
No previous imprisonment (*n* = 53)	6 (11.3)	47 (88.7)				
Gang affiliation (*n* = 26)	13 (50)	13 (50)	4.1	1.7–9.4	2.8	1.02–7.9
No gang affiliation (*n* = 197)	39 (19.8)	158 (80.2)				
Violent index offence (*n* = 82)	24 (30)	58 (70)	1.7	0.9–3.1		
Other conviction (*n* = 140)	28 (20)	112 (80)				
Sentence length ≥18 months^b^ (*n* = 70)	22 (31.4)	48 (68.6)	1.9	0.98–3.7		
Sentence length <18 months (*n* = 139)	27 (19.4)	112 (80.6)				
Clinical						
Depression (*n* = 70)	14 (20)	56 (80)	0.8	0.4–1.5		
No depression (*n* = 153)	38 (24.8)	115 (75.2)				
Psychosis^b^ (*n* = 24)	9 (37.5)	15 (62.5)	2.1	0.9–5.1		
No psychosis (*n* = 194)	43 (22.2)	151 (77.8)				
ADHD^b^ (*n* = 76)	22 (29)	54 (71)	1.5	0.8–2.9		
No ADHD (*n* = 144)	30 (20.8)	114 (79.2)				
Cluster B personality disorder^b^ (*n* = 143)	39 (27.3)	104 (72.7)	1.8	0.9–3.6		
No cluster B personality disorder (*n* = 75)	13 (17.3)	62 (82.7)				
Substance misuse (*n* = 77)	18 (23.4)	59 (76.6)	1.0	0.5–1.9		
No substance misuse (*n* = 146)	34 (23.3)	112 (76.7)				
Harmful alcohol use (*n* = 58)	15 (20.3)	59 (79.7)	0.8	0.4–1.5		
No harmful alcohol use (*n* = 165)	37 (24.8)	112 (75.2)				
PTSD (*n* = 26)	11 (42.3)	15 (57.7)	2.8	1.2–6.5	3.1	1.2–8.3
No PTSD (*n* = 197)	41 (20.8)	156 (79.2)			

Violence measured at 3-month follow-up. BME, Black and minority ethnic; ADHD, attention-deficit hyperactivity disorder; PTSD, post-traumatic stress disorder.

a. Adjusted for all other significant covariates in model.

b. Missing data.

Once all significant covariates were adjusted for, prisoners with PTSD were found to be over three times more likely to engage in violent behaviour at follow-up (adjusted odds ratio 3.1, 95% CI 1.2–8.3). The odds of perpetrating violence in custody were also found to significantly increase the longer individuals remained in custody (adjusted odds ratio 1.04, 95% CI 1.02–1.1), if they had been imprisoned before (adjusted odds ratio 3.7, 95% CI 1.3–10.4) or if they had gang affiliations (adjusted odds ratio 2.8, 95% CI 1.02–7.9). The odds of engaging in violent behaviour at follow-up were significantly reduced by older age (adjusted odds ratio 0.9, 95% CI 0.9–0.98).

### Mediation analysis

**Fig. 1:**
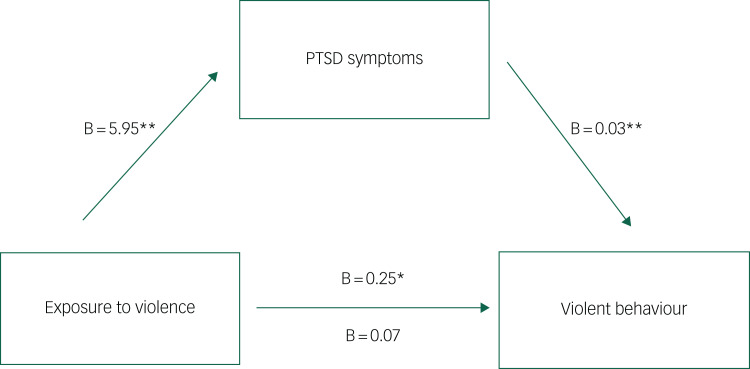
Individual mediation analysis of the relationship between interpersonal violence exposure and violent behaviour in prison, through PTSD symptom severity. Individual mediation analysis of total PTSD symptom severity on the pathway from interpersonal trauma exposure to violent behaviour. All effects represent beta coefficients adjusted for age and time at risk (days in custody). Values above the lines represent the c path (direct effect before mediation). Values below the lines represent the c’ path (direct effect after adjustment for the mediator). *P < 0.05, **P < 0.01. PTSD, post-traumatic stress disorder.

**Fig. 2:**
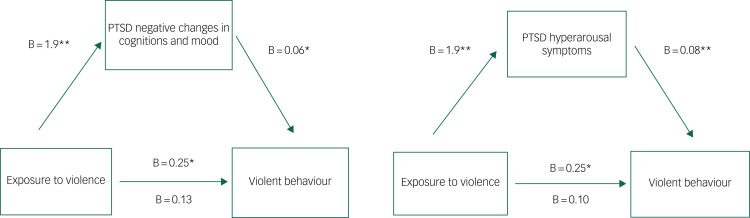
Individual mediation analysis of the relationship between interpersonal violence exposure and violent behaviour in prison, through PTSD symptom clusters. Individual mediation analyses of hyperarousal and negative changes in cognitions/mood PTSD symptoms on the pathway from interpersonal trauma exposure to violent behaviour in prison. All effects represent beta coefficients adjusted for age and time at risk (days in custody). Values above the lines represent the c path (direct effect before mediation). Values below the lines represent the c’ path (direct effect after adjustment for the mediator). *P < 0.05, **P < 0.01. PTSD, post-traumatic stress disorder.

Univariate logistic regression confirmed a significant association between previous exposure to interpersonal violence and violence in custody (odds ratio 1.2, 95% CI 1.1–1.4). Correlation analyses confirmed significant positive associations between interpersonal violence exposure, PTSD symptoms, anger and emotion dysregulation (see Figs 1 and 2, and Supplementary Material). Assessments of interaction between proposed exposure, mediating and confounding variables yielded no significant interaction effects.

Table 3 describes the results from individual mediation analysis. A significant indirect effect of exposure to interpersonal violence on violent behaviour in prison through total PTSD symptom severity was identified (B = 0.12, 95% CI 0.02–0.23), with PTSD symptoms mediating 69% of the total effect. This indirect effect remained after adjusting for the confounding effects of age and time at risk (B = 0.14, 95% CI 0.01–0.24), although the proportion of the total effect mediated decreased slightly to 59% (see Fig. 1).

**Table 3:** Individual mediation analysis for violent behaviour, with bootstrapped confidence intervals

	Unadjusted					Adjusted^a^				
Mediating variable	Total effect	Direct effect	Indirect effect	Bootstrapped 95% CI for indirect effect	Proportion mediated	Total effect	Direct effect	Indirect effect	Bootstrapped 95% CI	Proportion mediated
PTSD total	0.18	0.06	0.12	0.02–0.23	0.69	0.25	0.10	0.15	0.01–0.24	0.59
PTSD re-experiencing	0.18	0.11	0.07	−0.01 to 0.16	0.42					
PTSD avoidance	0.18	0.12	0.06	−0.02 to 0.16	0.35					
PTSD cognition/mood	0.18	0.07	0.11	0.008–0.20	0.59	0.25	0.13	0.12	0.02–0.21	0.47
PTSD hyperarousal	0.18	0.06	0.12	0.03–0.22	0.65	0.25	0.10	0.15	0.03–0.27	0.61
Anger	0.18	0.11	0.07	0.007–0.16	0.39	0.25	0.18	0.06	−0.03 to 0.11	0.26
Emotion dysregulation	0.19	0.14	0.05	−0.005 to 0.1	0.28					

PTSD, post-traumatic stress disorder.

a. Adjusted for age and time at risk (days in custody).

With respect to PTSD symptom subscales, the relationship between exposure to interpersonal violence and violent behaviour in prison was partially mediated by negative alterations in cognitions/mood (B = 0.11, 95% CI 0.03–0.22), and also by hyperarousal symptoms (B = 0.12, 95% CI 0.03–0.22) (see Fig. 2). In both models, the direct effects became non-significant, confirmed by bootstrapped confidence intervals. No mediation by re-experiencing (B = 0.08, 95% CI −0.01 to 0.2) or avoidance (B = 0.06, 95% CI −0.042 to 0.16) symptom clusters was identified. After adjusting for confounders, the indirect effects of exposure to interpersonal violence on violent behaviour through negative alterations in cognitions/mood (B = 0.12, 95% CI 0.01–0.22) and hyperarousal (B = 0.15, 95% CI 0.02–0.24) symptoms remained significant. Hyperarousal symptoms mediated a larger proportion of the total effect (61 *v*. 47%).

Results indicated a small yet significant indirect effect of interpersonal trauma on prison violence through anger (B = 0.07, 95% CI 0.007–0.16), but this effect was no longer significant after adjustment for confounders (B = 0.06, 95% CI −0.03 to 0.11) (see Table 3 or Supplementary Fig. 1). No mediation by emotion dysregulation was found; in this model, the direct and total effects remained significant, suggesting the presence of other hypothesised mediators not included in the analysis.^32^

## Discussion

In this prospective cohort study of sentenced male prisoners, we investigated whether PTSD represented an independent risk factor for prison violence. We further explored the mechanisms through which trauma exposure and its sequelae affect violent behaviour in custody, using mediation analyses.

Prisoners with PTSD were found to be at an increased risk of engaging in violent behaviour at 3-month follow-up in custody, after adjustment for sociodemographic and criminological risk factors. Such a finding is consistent with studies on military,^7^ community^10^ and offending populations.^11,33^ That PTSD emerged as a significant risk factor for prison violence, but other well-established risk factors for violence (such as psychosis or personality disorder) did not, is somewhat surprising.^19,34^ One possible explanation supported by previous research^35^ is that, compared with those with other disorders, prisoners with PTSD were less likely to be identified, monitored or treated by prison healthcare systems.

Second, particular PTSD symptom clusters were found to mediate the relationship between past exposure to interpersonal violence and violent behaviour in custody, representing longstanding shifts in thoughts, feelings and behaviour following a traumatic event. The hyperarousal and reactivity PTSD symptom cluster accounted for the largest proportion of the total relationship between trauma and violent behaviour, a finding supported by both empirical and theoretical research highlighting the role of hypervigilance and heightened arousal following trauma exposure on subsequent aggressive reactions to perceived threat.^36,37^

Evidence of mediation by the negative alterations in cognition and mood PTSD symptom cluster is supported by cognitive models of the effects of trauma on the development of negative appraisals.^38^ The development of negative cognitions, such as viewing the world as a dangerous place or believing that others cannot be trusted, are key aversive outcomes of trauma exposure, and several empirically supported theories of interpersonal aggression suggest that such appraisals increase the likelihood of responding to a perceived threat aggressively.^38,39^

Our third main finding was that the relationship between exposure to interpersonal trauma and prison violence was not found to be mediated by other core shared psychopathological features, namely anger or emotion dysregulation. This was surprising, given their strong links to both trauma and violent behaviour in past studies.^40,41^ It could be that self-rated trait anger alone was not sufficient in predicting violent behaviour in our investigation; although anger is often considered a prelude to aggression, not all individuals who report being angry will express that externally.^42^ Similarly, emotion dysregulation not subsumed by a diagnosis of PTSD may be more likely to be associated with self-destructive or self-injurious behaviour rather than aggression toward others, which may be more PTSD specific.^43,44^ Nonetheless, it is too early to conclude that PTSD (rather than anger or emotion dysregulation) is specifically linked to aggression, as such negative findings may also be a result of other methodological factors, such as the sampled population, follow-up period or the measure of prison violence used; further research clarifying these findings is therefore needed.

### Limitations

Our findings should be considered in light of some limitations. First, our moderately sized sample likely limited the power of the analyses to investigate predictors of violent and aggressive behaviour in custody, as indicated by some of the wide confidence intervals noted in analysis. As our investigation had multiple comparisons, future dedicated studies are needed to confirm our results.

Results from mediation analyses must also be interpreted with some caution. Our analysis was based on two time points, instead of the three typically assumed for mediation analysis. Effect sizes for mediation were often small, and direct effects became non-significant in some models, reflecting probable power issues. Limited power, coupled with high levels of conceptual overlap between our proposed mediators, also prevented us from conducting multiple mediation analysis with more than one mediator in each model. It is unlikely that the effects of interpersonal trauma exposure on prison violence are mediated via only one pathway, and examination of multiple mediators in the same model would allow for more precise analysis. Further replication in larger sample sizes that can support multiple mediation analysis is therefore needed.

The use of only prison records to determine the outcome of violent behaviour in custody, and self-report for some offence data, was a limitation because of reliance on a single record-based data source. Previous research has highlighted the improved validity gained through triangulation of outcome measurements through the incorporation of participant self-report and collateral informant information, as well as data from official records.^45^ It is possible that the absence of such corroborating measures may have led to an underestimation of the true prevalence of violent incidents, given that a substantial majority of assaults will go unreported.^46^ Our definition of violent behaviour included instances of verbal aggression and intimidation, which, although empirically justified, was broad and relatively heterogenous. Inconsistent and non-standardised record-keeping – common issues in prison record studies – also meant that we were unable to breakdown or stratify our violence outcome by type (e.g. verbal versus physical aggression) or severity, further impeding the specificity of our findings.

Finally, although many risk factors identified through literature searches were explored in analysis to establish potential confounding effects, there may have been other covariates that we could not account for, such as other measures of impulsivity, incarceration adjustment or social support.^34^ We also did not include assessment of autism spectrum disorder, other neurodevelopmental difficulties or traumatic brain injuries, which have demonstrated associations with prison violence and may therefore have influenced our findings.^47,48^ Our examination of pathways to violent and aggressive behaviour in prison was restricted to interpersonal trauma exposure, and a more detailed exploration of the impact of other forms of trauma (e.g. non-interpersonal traumas or child maltreatment) was considered beyond the scope of this investigation. Future studies may therefore wish to investigate differential associations between various forms of traumatisation and violence in custody.

### Implications and conclusions

The safety of prisons nationwide has been repeatedly called into question, highlighting a clear need for more effective assessment and intervention strategies to address violent behaviour in custody. The accurate identification of prisoners at risk of further violence is a challenging and complex task, and those who engage in physical violence are also at an increased risk of victimisation.^49^ Our study suggests that PTSD is an important independent risk factor for violent behaviour in prison settings, and that hyperarousal and negative cognitive and emotional appraisals are important mediators of the relationship between past exposure to violence and current institutional violence. PTSD is a treatable disorder, yet is often not adequately detected or treated by mental health professionals working in prison settings.^35^ Careful identification and treatment of these symptoms and this disorder may help to reduce both individual distress and rates of violent behaviour in prisons. Trauma-focused therapies have been found to be moderately effective in reducing PTSD symptoms among prison populations,^50^ and hyperarousal symptoms, particularly implicated in pathways to prison violence, have been shown to respond well to pharmacological treatment.^51^ Increasing resources for interventions aimed at treating post-traumatic stress symptoms could have a roll-on effect in reducing aggressive behaviour among offenders with PTSD, suggesting important avenues for prevention of future violence in the community as well as in prison settings.

The challenges inherent in trying to address trauma-related sequelae like PTSD in prison environments, which have an innate potential to re-traumatise, cannot be overlooked.^52^ The context of imprisonment is likely to increase the probability that its inhabitants will enter (or remain in) the ‘survival modes’ that theoretically link PTSD and aggression,^53^ and hyperarousal and hypervigilance may even be conceptualised as adaptive or protective coping strategies in response to the dangerous characteristics of many prison settings.^54^ Trauma-informed care initiatives in prison settings, which aim to minimise such re-traumatisation and promote values of safety and trust,^14,15^ merit additional critical evaluation.

## Data Availability

The data that support the findings of this study are available from the corresponding author, E.F.-I., upon reasonable request.

## Supplementary material

For supplementary material accompanying this paper visit http://doi.org/10.1192/bjo.2022.639. click here to view supplementary material
