# Supplementary material for: Trauma, post-traumatic stress disorder and violence in the prison population: prospective cohort study of sentenced male prisoners in the UK
Source: BJPsych Open. 2023 Mar 3;9(2):e47. doi: 10.1192/bjo.2022.639 (PMC10044336; doi:10.1192/bjo.2022.639)
Supplement: Supplementary file 1 [file bjosup.zip › S2056472422006391sup001.docx]

Exposure to Violence

Emotion Dysregulation

Violent Behaviour

B=2.56**

B= 0.02

B= 0.19*

B= 0.14

Exposure to Violence

Anger

Violent Behaviour

B= 3.2**

B= 0.02

B= 0.25*

B= 0.19

Supplementary Material Figure.. Individual mediation analysis of the relationship between interpersonal violence exposure and violent behaviour in prison – other trauma sequelae

Individual mediation analyses of emotion dysregulation and anger on the pathway from interpersonal trauma exposure to perpetration of violent behaviour in prison. Effects for emotion dysregulation represent unadjusted Beta coefficients. Effects for anger represent Beta coefficients adjusted for age and time at risk. Value above the line represents c path (direct effect prior to mediation). Value below line represents c’ path (direct effect after adjustment for the mediator). *p<0.05 **p<0.01
