# Supplementary material for: Trauma, post-traumatic stress disorder and violence in the prison population: prospective cohort study of sentenced male prisoners in the UK
Source: BJPsych Open. 2023 Mar 3;9(2):e47. doi: 10.1192/bjo.2022.639 (PMC10044336; doi:10.1192/bjo.2022.639)
Supplement: Supplementary file 1 [file bjosup.zip › S2056472422006391sup002.docx]

Supplementary Material

Figure 1 Supplementary Material

Recruitment flowchart of participants in study

**80 excluded following approach**

Insufficient English (n=39)

Lacked capacity/too unwell (n=11)

Risk concerns (n=4)

Did not meet criteria (n=9)

Could not take part (awaiting release or deportation) (n=17)

**237 took part in a clinical interview**

**N= 223 Final sample included in analysis**

5 withdrew consent during data collection

9 excluded due to too much missing data

9075 Prisoners admitted into custody during sampling period

**432 approached to participate**

**264 consented to take part**

**3477 Eligible to take part (Sentenced, age 18-55)**

**88 declined**

**27 did not complete an interview**

Transferred (n=6)

Released (n=7)

Withdrew consent (n=9)

Could not be located after repeated attempts (n=4)

Increased risk (n=1)

Excluded by researcher due to risk (n=1)

446 identified to approach

14 transferred/released prior to approach
