# Supplementary material for: Trauma, post-traumatic stress disorder and violence in the prison population: prospective cohort study of sentenced male prisoners in the UK
Source: BJPsych Open. 2023 Mar 3;9(2):e47. doi: 10.1192/bjo.2022.639 (PMC10044336; doi:10.1192/bjo.2022.639)
Supplement: Supplementary file 1 [file bjosup.zip › S2056472422006391sup003.docx]

Supplementary Table Correlations between proposed independent and mediating variables

|  | Variable | 2.  PCL5 | 3.  B | 4.  C | 5.  D | 6.  E | 7.  DERS | 8.  DAR |
| --- | --- | --- | --- | --- | --- | --- | --- | --- |
|  | Interpersonal Violence (IV) | 0.50*** | 0.42*** | 0.43*** | 0.45*** | 0.49*** | 0.34*** | 0.39*** |
|  | PCL5 Total | - | 0.88** | 0.77** | 0.94** | 0.91** | 0.64** | 0.52*** |
|  | PTSD Re-experiencing (B) |  | - | 0.70** | 0.75** | 0.71** | 0.57** | 0.40*** |
|  | PTSD Avoidance (C) |  |  | - | 0.68*** | 0.61*** | 0.48** | 0.37*** |
|  | PTSD Cognitions/Mood (D) |  |  |  | - | 0.82*** | 0.61** | 0.51*** |
|  | PTSD Hyperarousal (E) |  |  |  |  | - | 0.60** | 0.55*** |
|  | DERS Total |  |  |  |  |  | - | 0.67** |
|  | DAR Total |  |  |  |  |  |  |  |

PCL-5=Post-traumatic Checklist-DSM5; DERS= Difficulties in Emotion Regulation Scale; DAR= Dimensions of Anger Scale

*** p<0.001 **p<0.01 *p<0.05 Colour-coded to indicate strength of effect size: Grey= none; Blue= small; Green= medium; Orange= large.
